# Functional Annotation of *Caenorhabditis elegans* Genes by Analysis of Gene Co-Expression Networks

**DOI:** 10.3390/biom8030070

**Published:** 2018-08-03

**Authors:** Wei Liu, Ling Li, Yiruo He, Sen Cai, Wenjie Zhao, Hao Zheng, Yuexian Zhong, Shaobo Wang, Yang Zou, Zhenhua Xu, Yu Zhang, Wei Tu

**Affiliations:** 1 School of Life Sciences, Fujian Agriculture and Forestry University, Fuzhou 350002, China; 1150561002@fafu.edu.cn (L.L.); cs1159821786@gmail.com (S.C.); zhouzifang@mail.com (W.Z.); zhenghao@m.fafu.edu.cn (H.Z.); yuexianz@163.com (Y.Z.); 1160561008@fafu.edu.cn (S.W.); 1170539007@fafu.edu.cn (Y.Z.); 1170561006@fafu.edu.cn (Z.X.); 1170561001@fafu.edu.cn (Y.Z.); 2 School of Science and Engineering, The Chinese University of Hong Kong, Shenzhen 518172, China; andrewhe3@yahoo.com.cn; 3 Department of Molecular and Cellular Medicine, Texas A&M Health Science Center, College Station, TX 77843-1114, USA; tuwei1112003@163.com

**Keywords:** *Caenorhabditis elegans*, transcriptome, gene co-expression network, cuticle, hub gene

## Abstract

*Caenorhabditis elegans* (*C. elegans*) is a well-characterized metazoan, whose transcriptome has been profiled in different tissues, development stages, or other conditions. Large-scale transcriptomes can be reused for gene function annotation through systematic analysis of gene co-expression relationships. We collected 2101 microarray data from National Center for Biotechnology Information Gene Expression Omnibus (NCBI GEO), and identified 48 modules of co-expressed genes that correspond to tissues, development stages, and other experimental conditions. These modules provide an overview of the transcriptional organizations that may work under different conditions. By analyzing higher-order module networks, we found that nucleus and plasma membrane modules are more connected than other intracellular modules. Module-based gene function annotation may help to extend the candidate cuticle gene list. A comparison with other published data validates the credibility of our result. Our findings provide a new source for future gene discovery in *C. elegans*.

## 1. Introduction

High-throughput transcriptomics technology has been extensively applied to investigate the mechanisms of gene regulation. A promising strategy to find the gene functions of unknown genes is the gene co-expression method, which infers gene functions by similar gene expression patterns. This method has been used to explore the global, temporal, and spatial expression of *Caenorhabditis elegans* and its gene functions [1,2,3,4,5]. Early papers investigating these factors tried to elucidate the transcriptome in *C. elegans* with a relatively small sample size; for example, the 553 samples reported by Kim is the largest sample size to date [1]. Although recent studies have used state-of-the-art tiling arrays or RNA-Seq technologies, they focused on single genes or experimental conditions [6,7]. There are still genes that have been annotated with unknown function. For these genes, little is known about their biological function. To discover the tissue-, temporal-, or experimental condition-specific gene expression, large sample sizes are needed.

Microarray is a mature high-throughput method for genome-wide gene expression profiling. Thousands of microarray data have been deposited in public databases, however, most individual research uses differential expression analysis methods to find significant changes in expression, ignoring the inherent gene-gene expression correlation. Gene co-expression networks facilitate constructing a global view between genes [8]. Weighted gene co-expression network analysis (WGCNA) groups genes that have similar expression patterns across biological samples. In a gene co-expression network, a module is a subset of genes, whose expression patterns are similar to each other while different from genes in other modules. Usually, these genes are members from the same pathway or biological process. The whole transcriptome can be simplified into several modules, which allows us to look into biosystem components independently. Modules are more stable than individual genes in that the overall function of a module can remain the same while individual gene expression can be changed or replaced by other genes with similar redundant functions [9]. Furthermore, in a module, the importance of a gene can also be delineated by intramodule connectivity, which measures how correlated a gene is with all other module genes [10].

In this research, we applied WGCNA to publicly available *C. elegans* microarray data from different experimental conditions. Genome-scale modules of co-expressed genes with clear functional annotations were identified. Module-based qualitative analysis revealed that modules were associated with diverse biological functions. Module-based gene expression variation analysis suggested potential basal or conditional modules. Five modules that may correlate with molting were identified, and candidate cuticle genes were indicated. Those modules were also compared with previous publications to confirm the validity of our results.

## 2. Materials and Methods

### 2.1. Data Acquisition

Microarray datasets were obtained from the National Center for Biotechnology Information (NCBI) Gene Expression Omnibus (GEO) database under the platform number GPL200. For simplicity, only Affymetrix *C. elegans* Genome Array data were included. Briefly, 151 datasets with 2101 *C. elegans* samples were downloaded. Detailed information for these datasets is provided in Table S1.

### 2.2. Weighted Gene Co-Expression Network Analysis 

Microarray datasets were obtained from NCBI GEO database under the platform number GPL200. For simplicity, only Affymetrix *C. elegans* Genome Array Raw cel data were processed in Affymetrix Expression Console software (v1.4.1.46; Affymetrix, Inc., Santa Clara, CA, USA) using the MAS5 algorithm. Microarray data analysis was performed using R software (v3.1.2; R Foundation for Statistical Computing, Vienna, Austria) and Bioconductor WGCNA package (v1.51; R Foundation for Statistical Computing, Vienna, Austria; available from https://cran.r-project.org/src/contrib/WGCNA_1.51.tar.gz) [10]. Briefly, signed co-expression networks were constructed on the basis of 14,068 genes mapped from probe sets using the Brainarray Entrez Gene mapping file [11]. For each gene in the gene expression matrix, a pairwise Pearson correlation coefficient is computed, and an adjacency matrix is calculated by raising the correlation matrix to a power [12]. The power of 14 was chosen using the scale-free topology criterion. The weighted network was transformed into a network of topological overlap (TO)—an advanced co-expression measurement that measures not only the correlation of two genes, but also the extent of their shared correlations across the weighted network [12]. Genes were hierarchically clustered on the basis of their TO. Finally, co-expression gene modules were identified by the Dynamic Tree Cut algorithm [13]. Each module was summarized using singular value decomposition so that each module eigengene (ME) represented the first principal component of the module expression profiles [12]. Thus, ME explains the maximum amount of variation of the module expression levels, and is considered the most representative gene expression in a module. To construct the network of modules and identify meta-modules, the same process was applied to the above result. The parameters are power = 3, and minModuleSize = 2. The clustering used the hclust function in the WGCNA package. 

Connectivity for genes in each module was calculated by the softConnectivity function. Connectivity is a measurement of the sum of the gene expression correlation with all other genes. Genes with high connectivity in a specific module tend to be hub genes, which may play vital roles in module function. Module stability was tested by the average correlation between the original connectivity and the connectivity from half of the samples that were randomly sampled 1000 times. The process was run for every module.

### 2.3. Functional Annotation of the Modules 

Gene ontology (GO) enrichment for network modules were performed using the Database for Annotation, Visualization, and Integrated Discovery (DAVID) [14] with the background list of genes on the *C. elegans* genome array. In DAVID, an over-representation of a term is defined as a modified Fisher’s exact *p*-value with an adjustment for multiple tests using the Benjamini method. Other enrichments were performed by inputting a gene set into WormBase to find annotated terms that are over-represented using tissue enrichment analysis (TEA) and phenotype enrichment analysis (PEA) [15]. Modular genes enriched within chromosome regions were analyzed by the Positional Gene Enrichment analysis tool [16]. The stage specific module information was found by searching the NCBI PubMed database (www.ncbi.nlm.nih.gov/pubmed/) [17].

For gene expression variation analysis, gene expression relative standard deviation for each gene in a module were calculated and the average values for each module were provided.

### 2.4. Comparison of Gene Prediction Using Published Data

There are several early papers that tried to elucidate the transcriptome in *C. elegans* [1,3,6,18,19]. The module list and module gene list were compared. As for the WormNet prediction tool, which integrate heterogeneous genomics data into a single gene network for gene function prediction [18], we submitted our candidate gene into the tool, and observed if the predicted functions were similar to our module annotation.

## 3. Results and Discussion

### 3.1. A Gene Coexpression Network of C. elegans Was Successfully Constructed

**Figure 1:**
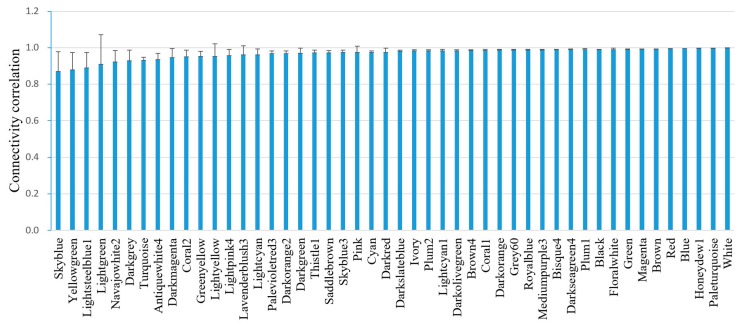
Correlation of intramodule connectivity for each module after 1000 instances of sampling (mean ± standard deviation (SD)). The connectivity values were calculated by sampling 1050 samples 1000 times randomly.

A total of 48 co-expressed gene modules were identified (Table 1). A representative network visualization was shown in Figure S1. For simplicity, only the top significant term was recorded. Functional annotation shows that these modules were associated with immune response, RNA processing, proteolysis, translation, signaling, embryo development, ion transport, reproduction, and many other biological processes. The module stability was tested by the correlation between the original connectivity and those calculated by 1000 half-sampled connectivity values for each module [20]. The correlations of connectivity were averaged for each module. All the modules have an average connectivity correlation larger than 0.8 (Figure 1). Among them, skyblue has the lowest module stability, while white has the highest module stability. These results indicate that the relationships between module genes were robust to the exclusion of 50% of the data.

There are several modules with same biological process. Ten modules are correlated with embryo development, three modules are correlated with ion transport, two modules are correlated with proteolysis, two modules are correlated with immune response, and two modules are correlated with reproduction. However, these were genes located on different chromosomes or expressed by different tissues. Further, tissue specific gene enrichment analysis revealed that these modules were associated with different tissues (Table 2).

### 3.2. Gene Expression Variation in Modules

**Figure 2:**
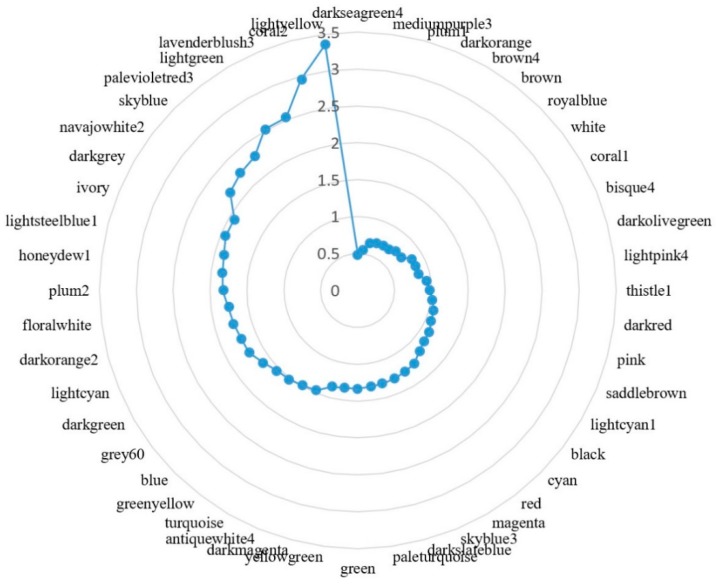
Radar chart showing module-based gene expression variation in *C. elegans*. The relative standard deviation (RSD) for each gene in a module was calculated, then the module gene expression variation was calculated by averaging the RSD of all genes. The blue dots represent the module RSD from highest (3.5) to lowest (0.5), reading anticlockwise.

As we have reduced the transcriptome data complexity by gene co-expression modules, we analyzed the gene expression variation at the module level. The gene level relative standard deviation (RSD) of gene expression was calculated, then module level RSD of gene expression was obtained by averaging the RSD of all genes (Figure 2). Sorting modules according to their RSDs, we can observe that the top 10 most stable modules include darkseagreen4 (mitochondrion), mediumpurple3 (mitochondrion), plum1 (mitochondrion), darkorange (mitochondrion), brown4 (embryo development ending in birth or egg hatching), brown (embryo development ending in birth or egg hatching), royalblue (embryo development ending in birth or egg hatching), white (ribosome), coral1 (embryo development ending in birth or egg hatching), and bisque4 (proteolysis). These modules are more related to housekeeping functions. The top 10 most variable modules include lightsteelblue1 (zinc ion binding), ivory (3′-untranslated region (UTR)-mediated mRNA destabilization), darkgrey (heterotrimeric G-protein complex), navajowhite2 (proteolysis), skyblue (nucleosome assembly), palevioletred3 (membrane), lightgreen (striated muscle dense body), lavenderblush3 (cul3-RING ubiquitin ligase complex), coral2 (structural constituent of cuticle), and lightyellow. Those modules are more related with stress response.

### 3.3. Modules That Correlate with Experimental Conditions

Modules can be relatively independent units which perform a biological function. Associating the modular gene expression with experimental conditions may help to discover a module’s functioning in specific conditions (Table S2). For example, amide-modified singlewalled carbon nanotube (a-SWCNT) treatment leads to the highest degree of coral2 module gene expression, which is a cuticle module. a-SWCNTs could cause retarded growth, reduced lifespan, and defective embryogenesis in worms [21]; acrylamide treatment induces the highest degree of red module gene expression, which is involved in cell shape regulation. Acrylamide could induces reversible de-phosphorylation of cytokeratins together with reversible filament aggregation [22]. This literature confirms our module GO result. The skyblue3 module has the highest expression in starved L1 wild-type worms, but the module has no significant GO annotation yet. A total of 56 of the 104 skyblue3 modular genes are annotated with hypothetical protein. These module genes may be involved in the starvation response.

### 3.4. Modules That May Correlate with Molting

*C. elegans* have a short life cycle of about three days at 22 °C. The cuticle protects *C. elegans* from environmental threats and allows growth by molting. It is synthesized five times, once in the embryo and subsequently at the end of each larval stage prior to molting [23]. Interestingly, we found five modules (coral2, darkslateblue, honeydew1, magenta, and paleturquoise) that are associated with the cuticle. Tissue enrichment analysis revealed that these modules may be associated with different tissue parts at different stages (Table 3). These modules are associated with collagen trimer and structural constituents of the cuticle. The module hub genes include collagen, unfolded protein response activated protein, and protease inhibitor, which may play roles in the cuticle structure [24].

To seek how these modules affect phenotypes, the transcription factor targeting enrichment was analyzed by WormExp based on curated and high-quality gene expression datasets [25]. These five module genes were submitted to WormExp. Paleturquoise were enriched with PMK-1 targets (*p* = 4 × 10^−16^). It has been shown that aging is associated with a decline in the activity of PMK-1 p38 mitogen-activated protein kinase pathway, which regulates innate immunity in *C. elegans* [26]. While, as a key component in barrier integrity, cuticle collagen could sense stress and participate in innate immunity [27]. The lifespan of the *pmk-1* mutant is reduced four-fold by wounding, but the effect is compromised by inhibiting bacterial proliferation [28]. Thus, the mechanism of increased pathogen susceptibility may due to the regulation of cuticle by PMK-1. Magenta and darkslateblue were both enriched with BLMP-1 targets based on genome-wide ChIP-seq in *C. elegans* (*p* = 4 × 10^−82^ and 2 × 10^−6^) [29]. Indeed, *blmp-1* mutants have a dumpy phenotype, a weak cuticle sensitive to oxidative stress, and show defective distal tip cells (DTC) migration [30]. 

To check whether the 48 modules were associated with specific chromosome regions, modular genes were subjected to Positional Gene Enrichment analysis. At a stringent *p* value (7 × 10^−7^), nine modules were identified to be associated with a specific chromosome region. Four of the molting related modules were associated with a chromosome region, including darkslateblue, honeydew1, magenta, and paleturquoise (Table S3).

### 3.5. Genes Function Annotation

To demonstrate the application of the gene co-expression module in gene function annotation, the coral2 module were selected as it has the smallest gene number. A total of 16 of the 34 modular genes are known collagen coding genes. Other modular genes encode include Ground-Like, sperm-coating protein (SCP)-Like extracellular protein, Cuticlin-1, and several hypothetical proteins. The hub gene is *col-2*, which is present only in dauer larva [31]. Another modular gene, *cut-1*, has been proven to code for a dauer-specific non-collagenous component of the cuticle [32]. These results suggest the possible dauer-specific role of the coral2 module. Six gene products were annotated with hypothetical protein. Their Entrez GeneIDs are 190357, 179082, 182552, 178567, 184159, and 187146. All these genes encode proteins containing transmembrane helices as predicted by TMPred (data not shown). Three of them contain a signal peptide at the N-terminus as predicted by SignalP (data not shown). These results indicate that those hypothetical proteins may be components of the extracellular cuticle [33].

### 3.6. Higher Order Module Organization

**Figure 3:**
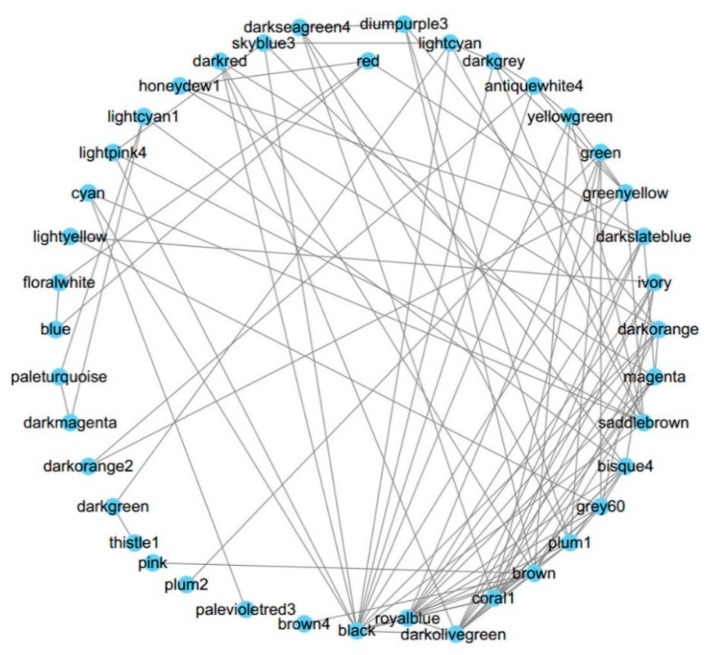
The network of modules. The higher order organization of the 48 modules was analyzed and the top 100 dense connections were visualized.

To observe the organization between these modules, the network of modules was also analyzed. These modules can form a higher-order network with 11 meta-modules (Figure 3). Global connectivity analysis shows that the top three highly connected modules are coral1, darkolivegreen, and black, whose cellular component annotations are nucleus (embryo development ending in birth or egg hatching) and plasma membrane (signal transducer activity), respectively. The three least connected modules are coral2, lavenderblush3, and navajowhite2, whose GO annotations are collagen trimer, Cul3-RING ubiquitin ligase complex, and proteolysis (Table S4). These results may indicate more complexity exists in the cell “brain” nucleus and the cell “gatekeeper” plasma membrane. Those modules with more specific intracellular functions are less connected (*p* = 2 × 10^−7^, *t* test). 

To check the relationship between these 11 meta-modules, a clustering diagram was plotted showing these modules can be divided into two main branches (Figure S2). The left branch modules are mainly associated with the GO embryo development ending in birth or egg hatching function, suggesting their distinct expression pattern from other processes.

### 3.7. Comparison with Previous C. elegans Networks

To validate our results, multiple publications results were compared. Kim constructed a gene expression map for *C. elegans*, and identified 43 sets of highly correlated genes [1]. They identified four collagen modules, five germline enriched modules, while we identified five collagen modules, and nine potential germline enriched modules. In their study, 14 of the 43 modules could not be annotated, while in our study only two remained unannotated. However, this may be due to there being fewer annotated genes in the database at that time.

Reinke analyzed the global profile of germline gene expression in *C. elegans* and found that sperm-enriched and germline-intrinsic genes are nearly absent from the X chromosome [3]. We identified nine modules that were annotated as embryo development ending in birth or egg hatching, but only the grey60 module genes were enriched in the X chromosome.

To compare our candidate gene list with those predicted by the online function prediction tools SPELL (v2.0.3; available: http://spell.caltech.edu:3000) [19] and WormNet (v3; available: http://www.functionalnet.org/wormnet) [18], the coral2 module had six unknown genes submitted [18,19]. Genes 179082 and 187146 had no prediction results in both tools. Genes 190357 and 184159 were predicted as body morphogenesis genes by WormNet. Genes 182552 and 178567 were predicted as genes with sensory perception of chemical stimulus by SPELL, which indicate their potential transmembrane location.

Another recent research finding is the transcriptome analysis of the developmental stages of the two sexes in *C. elegans*, which identified six major WGCNA modules [6]. All six modules were covered by our findings (Table S5). They summarized the modules with similar function, so only six major modules were retrieved. For example, they found only Mod4 with 1421 genes to be associated with the cuticle. We obtained five modules (Table 3) containing 912 genes that are associated with cuticle. When overlapping with those data, 518 shared genes were found (*p* < 5 × 10^−302^, hypergeometric test), which comprise about 57% of our module genes. 

A table containing all the module genes and their function annotation description information was provided in Table S6. For a better exploration of the module information provided in our analysis, a shiny-based web viewer was developed [34]. The tool site is available here: http://bioinformatics.fafu.edu.cn/shiny/sample-apps/celegans/

## 4. Discussion

Previous *C. elegans* transcriptome studies are limited by their sample size and specific conditions. Although recently scientists began to use state-of-art technologies, such as RNA-Seq and fluorescence activated cell sorting (FACS), to try and analyze the sex-, cell-, and stage-specific gene expression, 10 and 40 samples were profiled in the two studies [2,6]. This showed that a larger sample size may help to more robustly detect the gene co-expression modules in the human brain [13]. Here, we collected a compendium of *C. elegans* transcriptome data, the largest to date, and identified 48 co-expressed gene modules. These modules include genes for embryo development, cuticle, RNA processing, translation, ion transport and other biological processes. After identifying the gene modules, we further detected the associations between modules and experimental conditions, which may help to identify important modules in a specific condition. The five cuticle modules were subjected to gene prediction. Additionally, the higher order module organization analysis helps to illustrate the relationship between these functional units. Finally, our results were compared with previous studies to establish confidence. 

However, some limitations should be considered in the future studies. Technically, there are several parameters that can be adjusted according to data type and research purpose, but currently no standard guideline has been established for parameter setup. These parameters include multiple dataset integration, gene expression data normalization methods, similarity measures, and clustering methods. Biologically, the WGCNA presume that the relationships between genes are approximately linear, nevertheless, the reality is more complex [35]. Gene expression is not the final level in the biosystem, but many other levels of regulations makes the interpretation of gene co-expression network difficult. Although most of our modules have a clear functional annotation, we should be cautious when inferring the regulation relationships between genes. The WGCNA-derived gene network is undirected. More types of data, such as mutation, drug treatment, ChIp-Seq, and protein-protein interaction should be integrated to conclude the regulation. Thus, we mainly focus on gene function annotation and provide some potential transcription factor regulation information. In future, new algorithms should be applied to the emerging RNA-Seq data that accumulates. Conserved gene co-expression network can be obtained by comparing network from microarray and RNA-Seq. In sum, our analysis provides the module membership information and an overview of the *C. elegans* transcriptome, facilitating candidate gene screening in future experimental research for biologists.

## Figures and Tables

**Table 1:** Gene ontology (GO) and chromosome annotation of the identified 48 gene co-expression modules in *C. elegans*.

Module (No. of Genes)	Biological Process	Cellular Component	Molecule Function	Chromosome
Antiquewhite4 (35)	Ion transport (6 × 10^−7^)	Acetylcholine-gated channel complex (9 × 10^−5^)	Ion channel activity (2 × 10^−4^)	
Bisque4 (212)	Proteolysis (2 × 10^−9^)	Membrane raft (8 × 10^−10^)	Serine-type carboxypeptidase activity (3 × 10^−12^)	X (2 × 10^−5^)
Black (1173)	Ion transport (2 × 10^−16^)	Plasma membrane (5 × 10^−14^)	Signal transducer activity (1 × 10^−12^)	X (2 × 10^−13^)
Blue (930)	Regulation of cell shape (3 × 10^−25^)	Extrinsic component of cytoplasmic side of plasma membrane (8 × 10^−14^)	Protein kinase activity (1 × 10^−22^)	IV (1 × 10^−9^)
Brown (933)	Embryo development ending in birth or egg hatching (8 × 10^−41^)	Nucleus (3 × 10^−11^)	Protein binding (3 × 10^−8^)	I (1 × 10^−15^)
Brown4 (137)	Embryo development ending in birth or egg hatching (8 × 10^−14^)	Cytoplasm (2 × 10^−15^)	Protein binding (7 × 10^−5^)	III (4 × 10^−3^)
Coral1 (153)	Embryo development ending in birth or egg hatching (5 × 10^−11^)	Nucleus (1 × 10^−10^)	Protein binding (5 × 10^−2^)	I (3 × 10^−2^)
Coral2 (34)	Body morphogenesis (2 × 10^−2^)	Collagen trimer (2 × 10^−25^)	Structural constituent of cuticle (8 × 10^−26^)	
Cyan (219)	Axon guidance (2 × 10^−7^)	Axon (7 × 10^−3^)	Protein binding (3 × 10^−6^)	X (2 × 10^−20^)
Darkgreen (160)	Reproduction (8 × 10^−5^)	Cytoplasm (2 × 10^−2^)	Nucleotide binding (2 × 10^−4^)	III (1 × 10^−6^)
Darkgrey (136)	Neuropeptide signaling pathway (1 × 10^−9^)	Heterotrimeric G-protein complex (5 × 10^−5^)	Calcium ion binding (4 × 10^−4^)	X (2 × 10^−2^)
Darkmagenta (109)		Extracellular space (2 × 10^−4^)	Iron ion binding (3 × 10^−2^)	
Darkolivegreen (163)	Embryo development ending in birth or egg hatching (8 × 10^−3^)	Nucleus (1 × 10^−2^)	Protein binding (6 × 10^−4^)	III (8 × 10^−3^)
Darkorange (367)	Embryo development ending in birth or egg hatching (2 × 10^−9^)	Mitochondrion (6 × 10^−4^)		III (2 × 10^−9^)
Darkorange2 (221)	Innate immune response (4 × 10^−4^)			V (3 × 10^−4^)
Darkred (167)	Embryo development ending in birth or egg hatching (8 × 10^−11^)	P granule (8 × 10^−4^)		I (2 × 10^−3^)
Darkseagreen4 (41)	Nematode larval development (1 × 10^−7^)	Mitochondrion (6 × 10^−16^)	NADH dehydrogenase (ubiquinone) activity (4 × 10^−7^)	
Darkslateblue (127)	Endoplasmic reticulum unfolded protein response (1 × 10^−4^)	Collagen trimer (8 × 10^−34^)	Structural constituent of cuticle (9 × 10^−34^)	
Floralwhite (86)		Pseudopodium (3 × 10^−3^)		IV (1 × 10^−4^)
Green (602)	Embryo development ending in birth or egg hatching (3 × 10^−5^)	Nucleolus (6 × 10^−8^)	RNA binding (2 × 10^−6^)	I (4 × 10^−2^)
Greenyellow (389)	Nonmotile primary cilium assembly (1 × 10^−29^)	Ciliary basal body (2 × 10^−19^)	G-protein coupled receptor activity (5 × 10^−11^)	X (7 × 10^−4^)
Grey60 (522)	Embryo development ending in birth or egg hatching (8 × 10^−6^)	Nucleus (4 × 10^−14^)	Protein binding (9 × 10^−3^)	X (2 × 10^−18^)
Honeydew1 (44)	Collagen and cuticulin-based cuticle development (6 × 10^−4^)	Collagen trimer (5 × 10^−24^)	Structural constituent of cuticle (4 × 10^−27^)	
Ivory (95)	3′-UTR-mediated mRNA destabilization (2 × 10^−6^)	Nucleus (2 × 10^−6^)	mRNA 3′-UTR binding (1 × 10^−4^)	
Lavenderblush3 (46)		Cul3-RING ubiquitin ligase complex (4 × 10^−2^)		V (4 × 10^−2^)
Lightcyan (213)	Locomotion (6 × 10^−5^)	Axon (1 × 10^−4^)	Kinase activity (6 × 10^−5^)	X (8 × 10^−6^)
Lightcyan1 (98)	Innate immune response (2 × 10^-38^)	Membrane raft (4 × 10^−17^)	Carbohydrate binding (1 × 10^−5^)	IV (8 × 10^−3^)
Lightgreen (189)		Striated muscle dense body (8 × 10^−5^)		I (3 × 10^−3^)
Lightpink4 (49)	Maturation of LSU-rRNA (1 × 10^−3^)	Nucleolus (6 × 10^−10^)		
Lightsteelblue1 (98)			Zinc ion binding (3 × 10^−2^)	
Lightyellow (184)				II (4 × 10^−7^)
Magenta (592)	Molting cycle, collagen and cuticulin-based cuticle (2 × 10^−17^)	Extracellular region (1 × 10^−9^)	Structural constituent of cuticle (5 × 10^−7^)	X (4 × 10^−12^)
Mediumpurple3 (100)	Embryo development ending in birth or egg hatching (2 × 10^−15^)	Mitochondrion (7 × 10^−18^)	Threonine-type endopeptidase activity (6 × 10^−12^)	III (1 × 10^−2^)
Navajowhite2 (51)	Proteolysis (7 × 10^−3^)		Carbohydrate binding (1 × 10^−10^)	
Paleturquoise (114)	Lipid transport (2 × 10^−2^)	Extracellular region (1 × 10^−14^)	Structural constituent of cuticle (5 × 10^−11^)	X (3 × 10^−3^)
Palevioletred3 (55)		Membrane (4 × 10^−2^)		
Pink (306)		Endoplasmic reticulum (2 × 10^−2^)		III (9 × 10^−4^)
Plum1 (102)	ATP hydrolysis coupled proton transport (2 × 10^−11^)	Mitochondrion (8 × 10^−8^)	Proton-transporting atpase activity, rotational mechanism (4 × 10^−10^)	X (1 × 10^−2^)
Plum2 (65)				
Red (591)	Regulation of cell shape (2 × 10^−11^)	Integral component of membrane (5 × 10^−4^)	Phosphoprotein phosphatase activity (2 × 10^−8^)	IV (8 × 10^−4^)
Royalblue (292)	Embryo development ending in birth or egg hatching (4 × 10^−39^)	Nucleus (2 × 10^−31^)	Helicase activity (1 × 10^−7^)	III (3 × 10^−8^)
Saddlebrown (123)	Transforming growth factor beta receptor signaling pathway (2 × 10^−3^)	Axon (2 × 10^−6^)	Protein binding (3 × 10^−5^)	X (1 × 10^−5^)
Skyblue (128)	Nucleosome assembly (5 × 10^−6^)	Nucleosome (2 × 10^−6^)	Protein heterodimerization activity (5 × 10^−4^)	
Skyblue3 (104)				
Thistle1 (58)	Reproduction (1 × 10^−4^)	Nucleolus (4 × 10^−2^)		I (1 × 10^−2^)
Turquoise (3211)	G-protein coupled receptor signaling pathway (4 × 10^−205^)	Integral component of membrane (6 × 10^−296^)	G-protein coupled olfactory receptor activity (6 × 10^−139^)	V (4 × 10^−99^)
White (131)	Translation (3 × 10^−91^)	Ribosome (3 × 10^−122^)	Structural constituent of ribosome (4 × 10^−109^)	I (3 × 10^−3^)
Yellowgreen (104)	Ion transport (4 × 10^−5^)	Striated muscle dense body (1 × 10^−6^)	Actin binding (4 × 10^−4^)	

Note: Benjamini-adjusted Fisher’s exact test *p* values are given in brackets. NADH, Nicotinamide adenine dinucleotide; UTR, untranslated region; LSU, Large subunit; ATP, Adenosine triphosphate.

**Table 2:** Tissue enrichment for the 22 gene co-expression modules with the same GO BP annotation in *C. elegans*.

Module (No. Genes)	Biological Process	Tissue
Grey60 (522)	Embryo development ending in birth or egg hatching (8 × 10^−6^)	Caap
Darkred (167)	Embryo development ending in birth or egg hatching (8 × 10^−11^)	Psub1
Brown4 (137)	Embryo development ending in birth or egg hatching (8 × 10^−14^)	AWB
Darkorange (367)	Embryo development ending in birth or egg hatching (2 × 10^−9^)	Reproductive system, thermosensory neuron
Green (602)	Embryo development ending in birth or egg hatching (3 × 10^−5^)	Reproductive system, hermaphrodite distal tip cell
Darkolivegreen (163)	Embryo development ending in birth or egg hatching (8 × 10^−3^)	Reproductive system, pharyngeal interneuron
Royalblue (292)	Embryo development ending in birth or egg hatching (4 × 10^−39^)	Reproductive system, Capp
Mediumpurple3 (100)	Embryo development ending in birth or egg hatching (2 × 10^−15^)	Reproductive system, anal depressor muscle
Brown (933)	Embryo development ending in birth or egg hatching (8 × 10^−41^)	Reproductive system, Psub1
Coral1 (153)	Embryo development ending in birth or egg hatching (5 × 10^−11^)	Reproductive system, AVA
Darkorange2 (221)	Innate immune response (4 × 10^−4^)	AVA, pharyngeal interneuron
Lightcyan1 (98)	Innate immune response (2 × 10^−38^)	Intestine, outer labial sensillum, PVD
Black (1173)	Ion transport (2 × 10^−16^)	Ventral nerve cord, FLP, tail
Yellowgreen (104)	Ion transport (4 × 10^−5^)	Striated muscle
Antiquewhite4 (35)	Ion transport (6 × 10^−7^)	Pharyngeal interneuron, retrovesicular ganglion
Paleturquoise (114)	Lipid transport (5 × 10^−4^)	Cephalic sheath cell, hermaphrodite
Bisque4 (212)	Proteolysis (2 × 10^−10^)	Intestine, PVD
Navajowhite2 (51)	Proteolysis (6 × 10^−3^)	Male, reproductive system
Thistle1 (58)	Reproduction (3 × 10^−5^)	AVA
Darkgreen (160)	Reproduction (3 × 10^−5^)	Reproductive system

**Table 3:** Five modules that may correlate with molting in *C. elegans.*

Module	Tissue Enrichment (*p* Value)	Phenotype Enrichment (*p* Value)	Stage	Hub Gene
Coral2	Amphid socket cell (2 × 10^−4^)	Dumpy (3 × 10^−5^)	Dauer	*col-2*
Darkslateblue	Gonadal primordium (4 × 10^−7^)	Dumpy (7 × 10^−7^)	Embryo	ZK662.2
Honeydew1	Hyp7 syncytium (4 × 10^−10^)	Blistered (3 × 10^−6^)	L4	*col-138*
Magenta	Outer labial sensillum (2 × 10^−13^)	Molt variant (3 × 10^−20^), paralyzed (1 × 10^−11^)	L1	*abu-13*
Paleturquoise	Cephalic sheath cell (8 × 10^−27^)	Pathogen susceptibility increased (2 × 10^−6^)	Adult	C10G8.4

## Supplementary Materials

The followings are available online at http://www.mdpi.com/2218-273X/8/3/70/s1, Figure S1: *C. elegans* gene co-expression network. Each node represents a gene, and each line denotes the gene expression correlation between the two nodes. Figure S2: A clustering diagram representing the relationships between meta-modules (the higher order module); Table S1: *C. elegans* dataset used in our study; Table S2: Datasets that have the highest ME value in a specific module; Table S3: Modular genes enriched within chromosome regions by Positional Gene Enrichment analysis in *C. elegans*; Table S4: Module network and connectivity; Table S5: Our module results compared with reference [6]; Table S6: Module genes and connectivity and their function annotation.
