# Supplementary material for: Functional Annotation of Caenorhabditis elegans Genes by Analysis of Gene Co-Expression Networks
Source: Biomolecules. 2018 Aug 3;8(3):70. doi: 10.3390/biom8030070 (PMC6163173; doi:10.3390/biom8030070)
Supplement: Supplementary file 1 [file biomolecules-08-00070-s001.zip › Figures Supplementary.pdf]

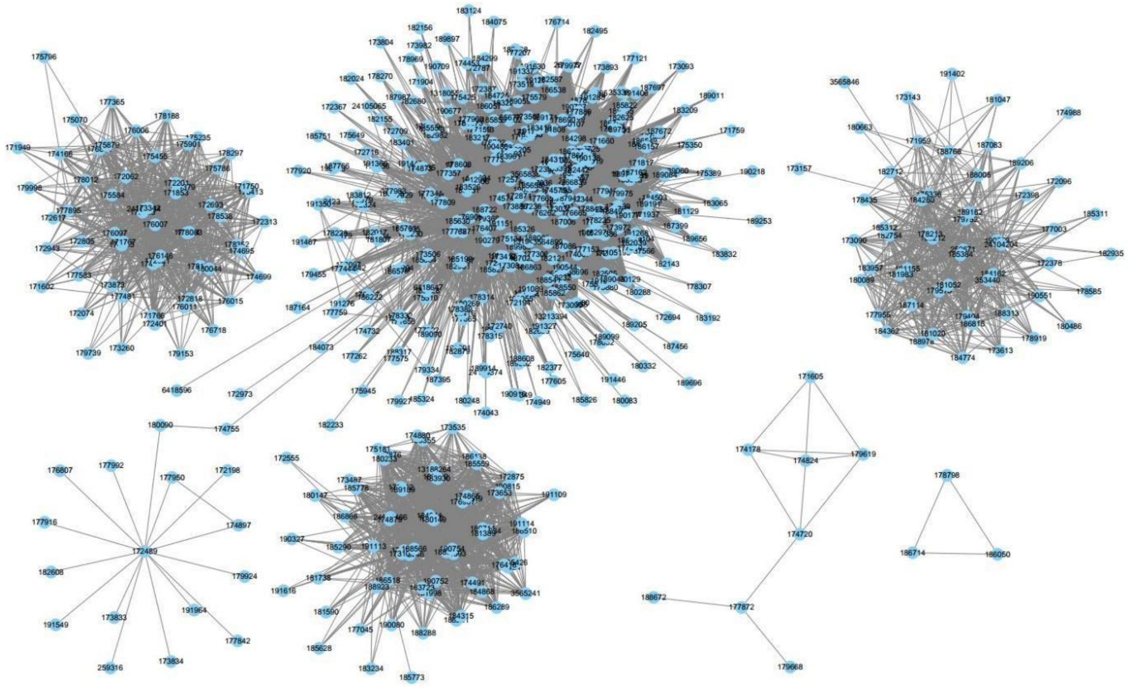

Figure S1. *C. elegans* gene co-expression network. Each node represents a gene, and each line denotes the gene expression correlation between the two nodes.

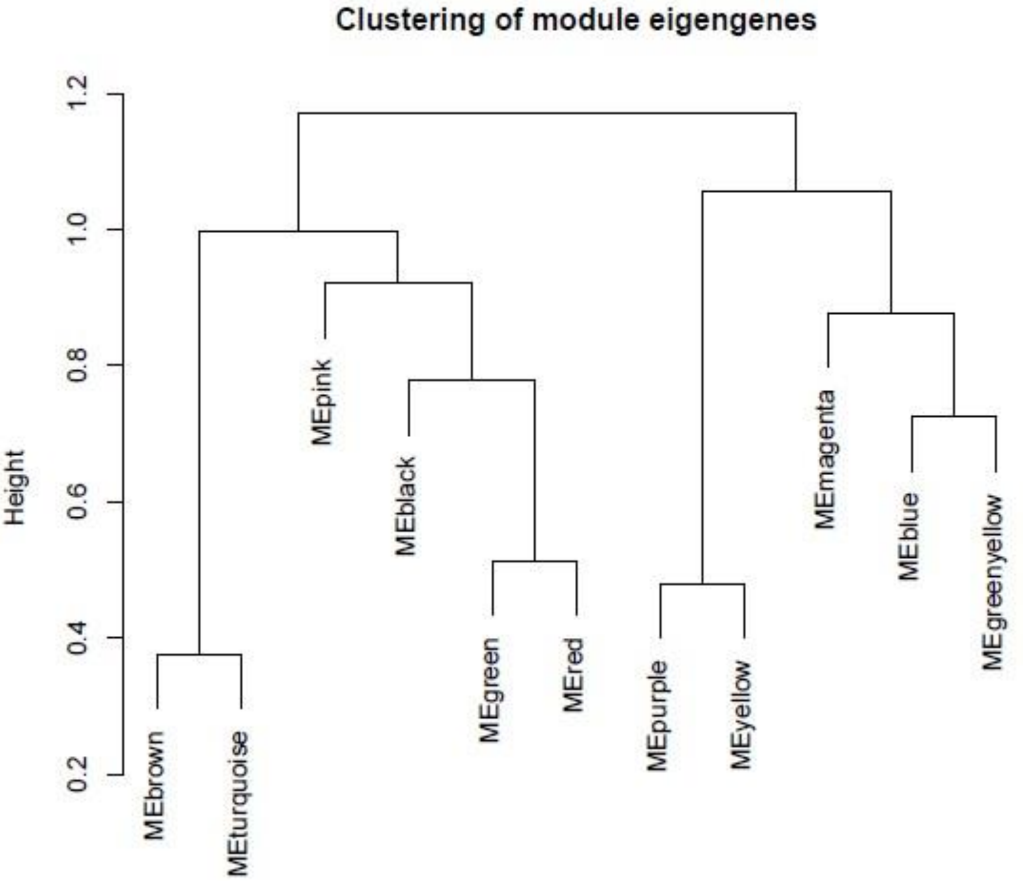

Figure S2. A clustering diagram representing the relationships between meta-modules (the higher order module).
